# Supplementary material for: Identifying individuals with high risk of Alzheimer’s disease using polygenic risk scores
Source: Nat Commun. 2021 Jul 23;12:4506. doi: 10.1038/s41467-021-24082-z (PMC8302739; doi:10.1038/s41467-021-24082-z)
Supplement: Supplementary file 1 — Supplementary Information [file 41467_2021_24082_MOESM1_ESM.pdf]

Supplemental Material

Supplementary Note 1: Quality-Control analysis and data harmonisation

SNPs were removed with Minor Allele frequency (MAF) <0.01; Hardy-Weinberg equilibrium ($p \leq 10^{-6}$); with missing data proportion >5% in UKBB, 1000 Genomes, HipSci and >2% in ADNI, ROSMAP, MSBB, MAYO; SNPs with poor accuracy of imputation (INFO ≤ 0.4) in UKBB and (INFO ≤ 0.7) in HipSci; or with non-autosomal location. Samples were removed on the basis of call rate <98%; heterozygosity (HET > ±0.1); relatedness based on identity by descent with PI_HAT >0.2 in 1000 Genomes, UKBB, HipSci, ADNI and PI_HAT >0.22 for ROSMAP, MSBB, MAYO; list of recommended sample exclusions from AMP-AD portal for ROSMAP, MSBB, MAYO. Studies were merged with 1000 Genomes data to compute principal components (PC) and individuals were removed that did not cluster near 1000 Genomes European. More informatics can be found in Supplementary Table 1.

To gain more power we combined and harmonised ADNI, ROSMAP, MSBB and MAYO studies, removed overlapping samples that were used in Kunkle GWAS study [2], leaving 271 AD cases and 278 controls with 6,077,045 SNPs for the further analysis (see details in Supplementary Table 2). This sample was also combined with the European 1000 Genomes sample to enable PRS standardisation against a population cohort. To adjust for population stratification principal components were computed in the full merged datasets (i.e. ADNI+ROSMAP+MSBB+MAYO+1000G); pairwise PCs were plotted and those which displayed heterogeneity were used to adjust the PRS. Eight PCs were used to account for population structure in the case-control sample (ADNI+ROSMAP+MSBB+MAYO+1000G) and twenty PCs were used in the (HipSci+1000G+UKBB) data.

Supplementary Table 1: *Description of studies (genetics).*

**Study**	**Genotypes available N samples**	**Study design**	**N variants Genotyping/ Imputation**	**After QC: N samples**	**After QC: N variants**
1000G (EUR)	503	Population	80,000,000 WGS	503	9,047,395
UK Biobank	500,000	Population	35,884,914 HRC imputed SNPs	443,018	7,654,308
HipSci	1254	Population	6,216,949 HRC imputed SNPs	1228	5,454,509
ADNI	770	Case-control	42,732,452 WGS	293	7,808,548
ROSMAP	1196	Case-control	43,440,340 WGS	52	8,717,089
MAYO	349	Case-control	27,537,089 WGS	121	8,717,089
MSBB	349	Case-control	35,445,106 WGS	83	8,717,089

Legend: *Genetic information available by study: number of individuals with genetic profile, number of variants (whole genome sequenced (WGS) or imputed using the Haplotype Reference Consortium (HRC)), study design followed by number of samples and variants retained after the Quality-Control (QC) analysis described in Supplementary Section 1.*

Supplementary Table 2: *Definition of AD case-control status in ADNI, ROSMAP, MAYO, MSBB that overlaps with genetic data and their APOE distribution.*

**Study**	**Diagnostic criteria**	**Thresholds for Case-control definition**	**Number of cases and controls**	**AGE Mean (min and max)**	***APOE* distribution in (ε2ε2/ε2ε3/ε2ε/ε3 ε3/ε3ε4/ε4ε4)**
ADNI	Clinical diagnosis at last assessment: 1-stable control; 2-stable MCI; 3-stable AD; 4-conversion control to MCI; 5-conversion MCI to AD; 7-conversion MCI to control;8- AD to MCI.	1-Control 3-AD	126/167	Age at the last interview 77.3(55.6, 94.5)	0/27/2/131/105/28
ROSMAP	Final consensus diagnosis: 1-control; 2-MCI no other cause of CI; 3-MCI with another cause of CI; 4- AD-no other cause of CI; 5-AD with another CI; 6-Other dementia.	1 –control 4,5 –AD case	31/21	Age at death 85.4(70.4,90+)	0/4/2/30/13/3
MAYO	Clinical diagnosis: AD, Progressive Supranuclear Palsy, Pathological Aging, Control	Control-control AD-AD case;	68/53	Age at death 82.2(58, 90+)	0/14/1/64/35/7
MSBB	Clinical Dementia Rating: 0-no dementia; 0.5-questinable dementia, 1-mild dementia; 2-moderate dementia; 3-severe dementia, 4-profound dementia, 5-terminal dementia	0, 0.5-Control 3,4,5 –AD case*	46/37	Age at death 82.5(61,90+)	0/10/1/42/30/0

Legend: Phenotypic information available by study: clinical diagnosis criteria; threshold that was used for definition of AD cases and cognitively normal controls; number of cases and controls retained in the analysis; mean (minimum and maximum) of age and APOE distribution. Only CDR was available to us in MSBB study, which does not distinguish between different types of dementia, therefore there is the potential for AD to be misdiagnosed. Abbreviations: AD-Alzheimer's' Disease; MCI-Mild Cognitive Impairment; CI-Cognitive Impairment.

Supplementary Table 3: *PRS prediction accuracy for the AD case-control dataset using different p-value thresholds, methods to model APOE and different LD-clumping thresholds (r^2).*

pT	r^2	PRS				PRS.no.APOE				PRS.AD		
		N SNPs	AUC (%)	R^2	OR (95% CI)	N SNPs	AUC (%)	R^2	OR (95% CI)	AUC (%)	R^2	OR (95% CI)
5e-8	0.01	33	66.8	0.13	2.0 (1.6, 2.4)	16	56.0	0.02	1.2 (1.0, 1.5)	71.2	0.19	2.4 (2.0, 3.0)
1e-5		80	68.4	0.15	2.1 (1.7, 2.6)	58	55.4	0.01	1.2 (1.0, 1.4)	71.4	0.19	2.4 (2.0, 3.0)
0.1		33493	63.1	0.08	1.7 (1.4, 2.0)	33435	60.5	0.06	1.5 (1.3, 1.8)	74.0	0.23	2.7 (2.2, 3.4)
0.5		71366	60.5	0.05	1.5 (1.3, 1.8)	71308	58.9	0.04	1.4 (1.2, 1.7)	73.1	0.22	2.6 (2.1, 3.2)
5e-8	0.001	19	67.6	0.14	2.1 (1.7, 2.5)	15	55.0	0.01	1.2 (1.0, 1.4)	71.2	0.19	2.4 (2.0, 3.0)
1e-5		63	69.0	0.16	2.2 (1.8, 2.7)	58	55.3	0.01	1.2 (1.0, 1.4)	71.4	0.19	2.4 (2.0, 3.0)
0.1		10176	60.6	0.04	1.5 (1.2, 1.7)	10163	57.0	0.02	1.3 (1.1, 1.5)	71.6	0.19	2.4 (2.0, 3.0)
0.5		17031	59.9	0.04	1.4 (1.2, 1.7)	17023	57.6	0.03	1.3 (1.1, 1.6)	72.0	0.20	2.5 (2.0, 3.0)

Legend: *PRSs(C+T) were calculated on a case-control cohort (271 clinically defined AD cases and 278 cognitively normal controls) using Kunkle et al. (2019) summary statistics for pT≤ 5e-8, 1e-5, 0.1, 0.5 LD-pruned SNPs and APOE(ε2+ε4). The number of SNPs (NSNPs) in each risk score are reported. Three models were considered: PRS calculated on the full summary statistics; PRS.no.APOE where the APOE region was excluded (chr19:44.4-46.5Mb); PRS.AD which is calculated as a weighted sum of PRS.no.APOE and APOE(ε2+ε4), where APOE effects were weighted with effect sizes (B(ε2)=-0.47 and B(ε4)=1.12) as in Kunkle et al (2019). Prediction was estimated in terms of AUC, R^2 and OR with 95% Confidence Intervals (CI).*

Supplementary Table 4: *Prediction Accuracy and Number of Extremes Across Different PRS Methods.*

Method	Model	AUC (%)	R2	OR (95% CI)	No of Positive Extremes at 2SD (N Cases)	No of Negative Extremes at 2SD (N Controls)
PRS (C+T)	ORS.full	69.4	0.162	10 (1, 75)	38 (33)	5 (3)
	ORS.no.APOE	56.7	0.016	2 (0.3, 9)	12 (6)	11 (7)
PRSice	ORS.full	69.0	0.146	11 (1, 70)	32 (26)	7 (5)
	ORS.no.APOE	56.5	0.015	2 (0.4, 12)	11 (6)	11 (7)
LDpred-inf	ORS.full	67.3	0.123	4 (0.9, 23)	33 (24)	8 (5)
	ORS.no.APOE	57.0	0.019	10 (0.5, 205)	19 (9)	5 (5)
PRS-CS	ORS.full	68.2	0.134	17 (4, 68)	39 (32)	19 (15)
	ORS.no.APOE	55.3	0.011	3 (0.4, 16)	18 (9)	7 (5)
LDAK	ORS.full	65.1	0.095	23 (2, 236)	24 (19)	7 (6)
	ORS.no.APOE	55.5	0.013	9 (0.9, 91)	16 (9)	8 (7)
SBayesR	ORS.full	54.5	0.009	4 (0.6, 26)	15 (8)	9 (7)
	ORS.no.APOE	55.4	0.012	3 (0.6, 18)	17 (10)	10 (7)
PRS (C+T)	PRS .full	64.9	0.093	32 (6, 180)	22 (19)	18 (15)
	PRS.no.APOE	61.3	0.058	32 (6, 182)	20 (17)	20 (17)
	PRS.AD	74.1	0.236	124 (6, 2707)	36 (33)	6 (6)
PRSice	PRS.full	64.0	0.083	18 (4, 88)	21 (18)	20 (15)
	PRS.no.APOE	60.9	0.053	36 (6, 202)	20 (17)	22 (19)
	PRS.AD	73.9	0.231	124 (6, 2707)	36 (33)	6 (6)
LDpred-inf	PRS.full	62.5	0.074	12 (3, 45)	30 (25)	24 (17)
	PRS.no.APOE	59.7	0.049	10 (3, 36)	30 (24)	25 (18)
	PRS.AD	73.6	0.226	113 (6, 2243)	41 (36)	8 (8)
PRS-CS	PRS.full	66.7	0.116	23 (4, 125)	30 (22)	19 (17)
	PRS.no.APOE	59.9	0.042	7 (2, 25)	33 (20)	22 (18)
	PRS.AD	73.3	0.220	82 (4, 1661)	39 (34)	6 (6)
LDAK	PRS.full	62.5	0.062	5 (1, 16)	32 (23)	20 (13)
	PRS.no.APOE	60.4	0.046	5 (1, 18)	30 (22)	17 (11)
	PRS.AD	73.4	0.217	21 (3, 148)	38 (34)	7 (5)
SBayesR	PRS.full	60.7	0.047	4 (1, 14)	19 (13)	19 (12)
	PRS.no.APOE	60.8	0.047	3 (1, 12)	19 (12)	17 (11)
	PRS.AD	73.9	0.227	69 (3, 1431)	39 (34)	5 (5)

Legend: *Prediction accuracy and number of PRS extremes are presented for 6 PRS approaches: PRS(C+T), PRSice, LDpred-Inf, PRS-CS, LDAK, SBayesR for two p-value thresholds pT≤1e-5 (ORS) and pT<0.1 (PRS). PRS was computed in the case-control sample (271 cases and 278 controls) and weighted with effect sizes from Kunkle et al. 2019. Three models were assessed within each method a) ORS.full/PRS.full calculated on the full summary statistics b) ORS.no.APOE/PRS.no.APOE where the APOE region was excluded (chr19:44.4-46.5Mb) c) PRS.AD calculated as a weighted sum of PRS.no.APOE and*

APOE(ε2+ε4). PRS extremes were defined as individuals with a score exceeding ±2 SD from the population mean (1000 Genomes). Prediction accuracy was assessed with AUC(%), R^2, OR with 95% Confidence Intervals (calculated on extremes only), total number of positive extremes (number of cases (Ncases) in the positive tail), total number of negative extremes (number of controls (Ncontrols) in negative tail).

Supplementary Table 5. *PRS distribution parameters in cases and controls for in-sample and population-based standardisation in the case-control data*

**Distribution Parameters**		**In-sample standardisation**		**Population-based standardisation**	
		**Cases**	**Controls**	**Cases**	**Controls**
ORS	Mean	0.352	-0.343	0.478	-0.335
	SD	1.063	0.798	1.242	0.931
ORS.no.APOE	Mean	0.111	-0.108	0.180	-0.032
	SD	0.927	1.057	0.900	1.027
PRS	Mean	0.265	-0.258	0.385	-0.193
	SD	0.989	0.943	1.092	1.041
PRS.no.APOE	Mean	0.210	-0.205	0.320	-0.136
	SD	0.988	0.971	1.089	1.070
PRS.AD	Mean	0.426	-0.415	0.630	-0.370
	SD	1.009	0.796	1.200	0.946

Legend: *PRS(C+T) distribution parameters (mean and standard deviation) for five models: ORS (pT≤1e-5), PRS (pT≤0.1) with and without APOE region and PRS.AD (pT≤0.1) standardised a) within sample (271 cases and 278 controls) and b) on 1000 Genomes (503 individuals).*

Supplementary Fig. 1. *Title: Prediction accuracy and variance explained by APOE, ORS and PRS models in younger and older study participants; simulation study.*

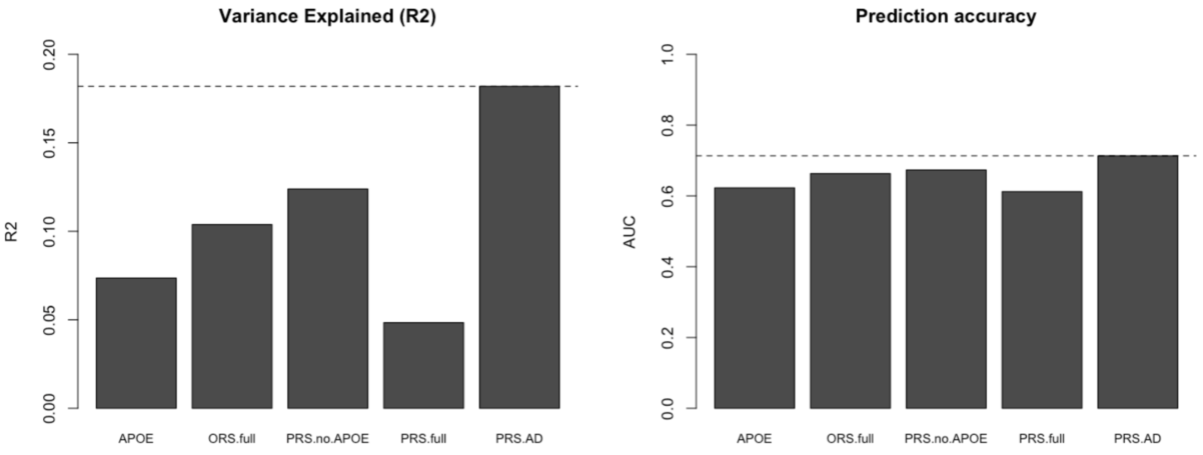

Legend: *Prediction accuracy and variance explained by APOE, ORS ($pT \leq 1e\text{-}5$) and PRS ($pT \leq 0.1$) accounting for differential distribution of ε4 alleles in younger and older study participants in the simulation study when controls are sampled from the general population.*

Supplementary Fig. 2. *Title: AD PRSs distributions for UK Biobank (UKBB) and 1000 Genomes (EUR).*

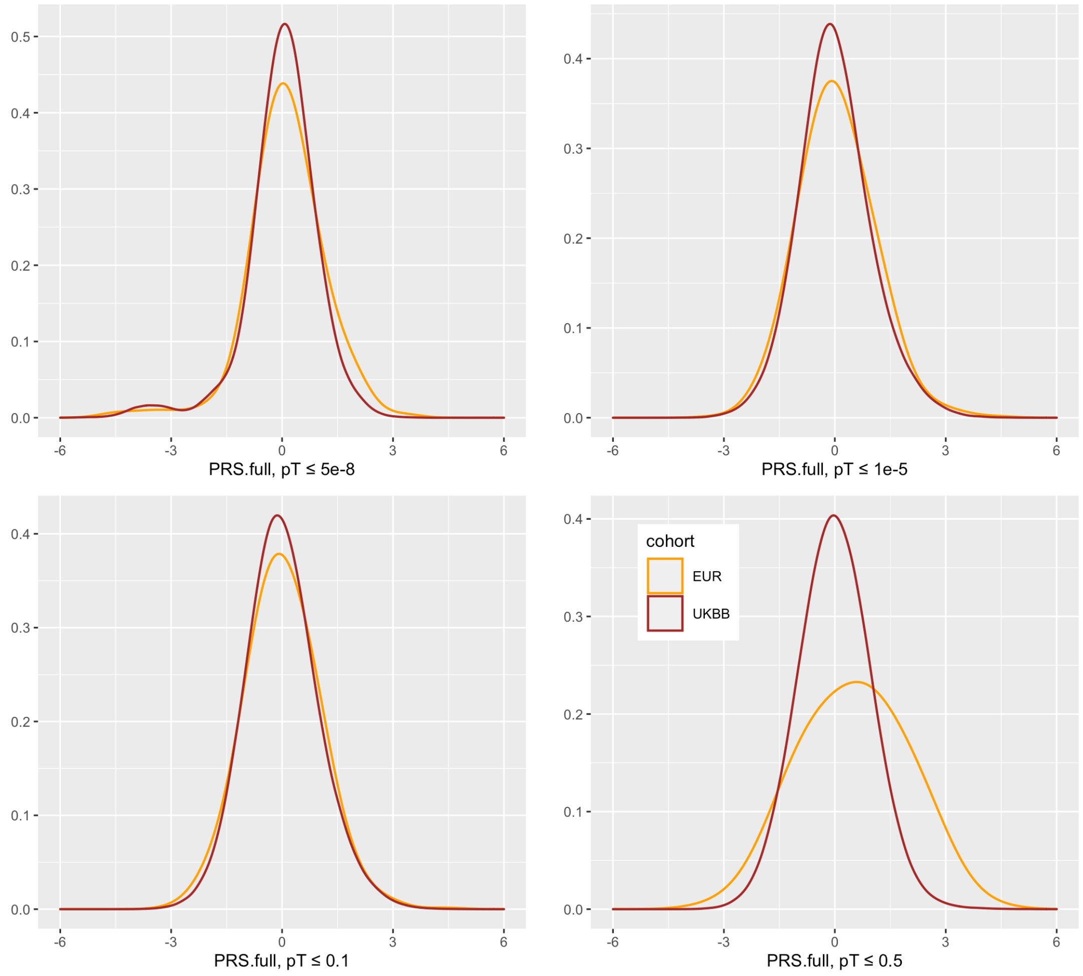

Legend: *PRS(C+T) distributions for UKBB (brown) and 1000 Genomes (orange) calculated on Kunkle et al. (2019) summary statistics for pT≤5e-8, 1e-5, 0.1, 0.5 LD pruned SNPs. PRS distributions have not been adjusted for PCs. PRS=Polygenic risk score; C+T=Clumping and thresholding; pT= p-value threshold; LD=Linkage disequilibrium; SNP=Single nucleotide polymorphism; PC=Principal component.*

Supplementary Fig. 3. *Title: PRS.AD distributions for case-control dataset using in-sample and population standardisations.*

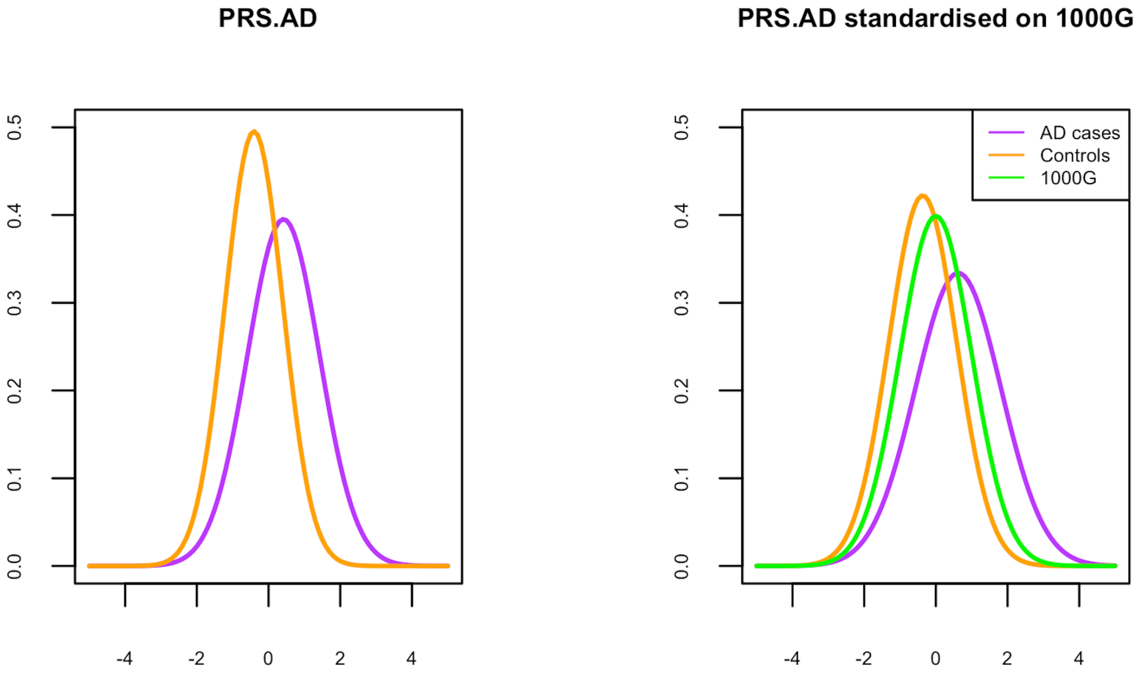

Legend: *PRS.AD distributions for case-control dataset (271 cases and 278 controls) that are standardised in-sample (left) and on 1000 Genomes (right). PRS.AD is calculated as a weighted sum of PRS.no.APOE (excluding SNPs in APOE region) and APOE(ε2+ε4). The distribution parameters are estimates from Supplementary Table 4. Purple line is the distribution of cases, orange line is the distribution of controls and the green line is the distribution of the population sample. PRS=Polygenic risk score; AD=Alzheimer's Disease; SNP=Single nucleotide polymorphism.*

Supplementary Fig. 4. *Title: Spider-plot for number of shared PRS extremes across six PRS approaches for ORS and PRS.*

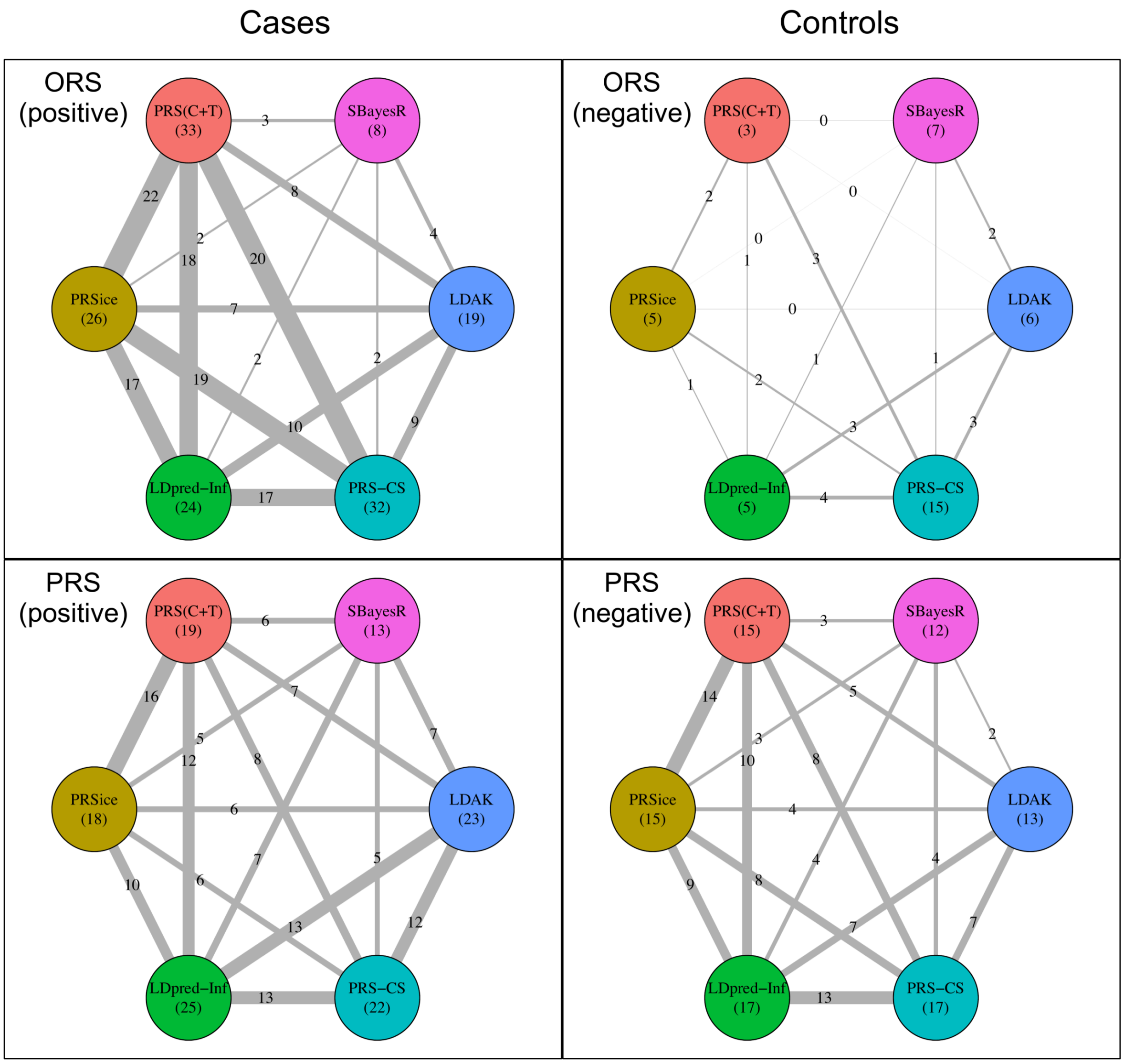

Legend: *Pairwise network visualisation plot showing the number of common individuals identified by each pairwise combination of the 6 PRS approaches (PRS(C+T), PRSice, LDpred-Inf, PRS-CS, LDAK, SBayesR; red, yellow, green, teal, blue, pink respectively). The total number of correctly identified extremes (cases in positive tail, controls in negative tail) are shown in brackets under the corresponding PRS approach. Line widths show the number of shared identifications, also depicted numerically. Rows are ORS.full (pT≤ 1e-5) (top) and PRS.full (pT≤ 0.1) (bottom). Columns are number of cases in the positive tail (left) and*

controls in the negative tail (right). ORS=Oligogenic risk score; PRS=Polygenic risk score; C+T=Clumping and thresholding; pT=p-value threshold.

Supplementary Fig. 5. *Title: Map of top and bottom PRS.no.APOE (p≤0.1) extremes in e33 individuals from A) PRS(C+T) B) PRSice, C) LDpred-Inf, D) PRS-CS, E) LDAK and F) SBayesR across different PRS methods. The y-axis displays the PRS standardised against the 1000 Genome cohort.*

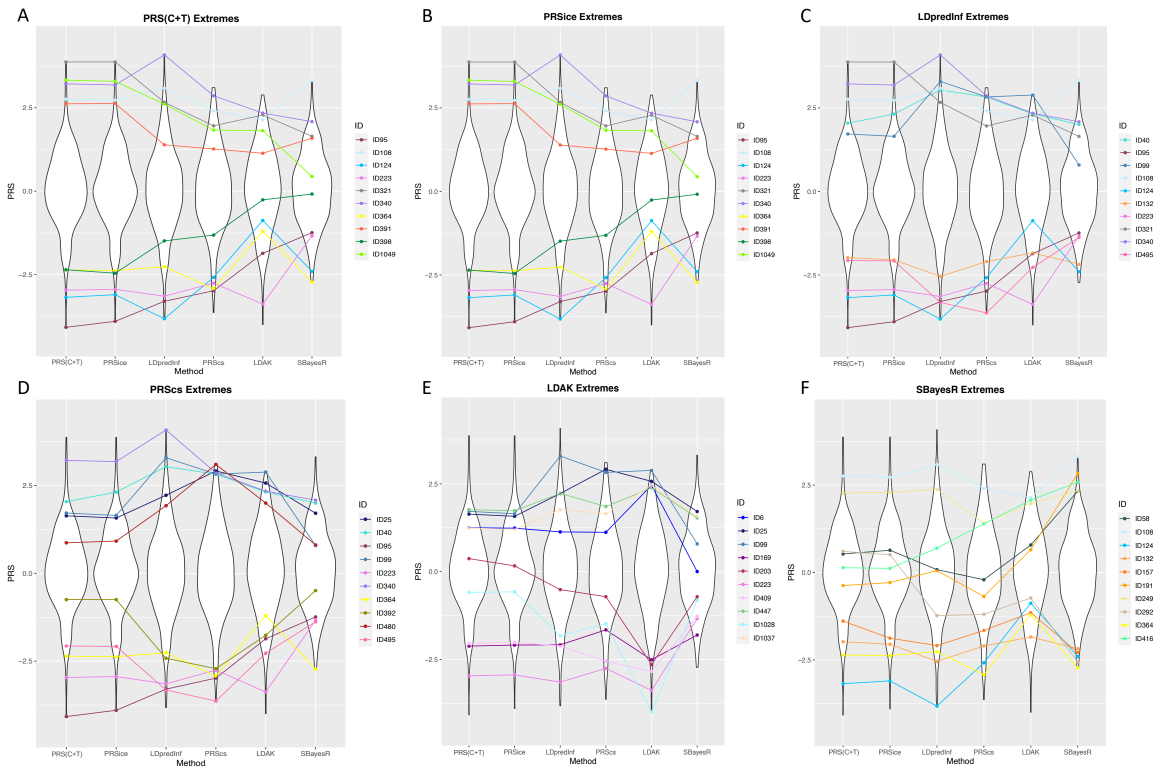

Legend: A map of the top 5 and bottom 5 extremes selected by each different PRS method, and how these extremes compare across the different methods. Violins show the PRS.no.APOE distributions standardised against the 1000 Genome cohort. PRS.no.APOE excludes SNPs in the APOE region. IDs are anonymised and the colour scheme is consistent across the 6 plots to highlight extremes selected by multiple PRS approaches.

ORS=Oligogenic risk score; PRS=Polygenic risk score; SNP=Single nucleotide polymorphism.
