# Supplementary material for: Identifying individuals with high risk of Alzheimer’s disease using polygenic risk scores
Source: Nat Commun. 2021 Jul 23;12:4506. doi: 10.1038/s41467-021-24082-z (PMC8302739; doi:10.1038/s41467-021-24082-z)
Supplement: Supplementary file 3 — Reporting Summary [file 41467_2021_24082_MOESM3_ESM.pdf]

nature research

Corresponding author(s): *Double-blind peer review submissions: write DBPR and your manuscript number here instead of author names.*

Last updated by author(s): *YYYY-MM-DD*

Reporting Summary

Nature Research wishes to improve the reproducibility of the work that we publish. This form provides structure for consistency and transparency in reporting. For further information on Nature Research policies, see our Editorial Policies and the Editorial Policy Checklist.

Statistics

For all statistical analyses, confirm that the following items are present in the figure legend, table legend, main text, or Methods section.

n/a	Confirmed	
☐	☒	The exact sample size (*n*) for each experimental group/condition, given as a discrete number and unit of measurement
☐	☒	A statement on whether measurements were taken from distinct samples or whether the same sample was measured repeatedly
☐	☒	The statistical test(s) used AND whether they are one- or two-sided<br>*Only common tests should be described solely by name; describe more complex techniques in the Methods section.*
☐	☒	A description of all covariates tested
☒	☐	A description of any assumptions or corrections, such as tests of normality and adjustment for multiple comparisons
☐	☒	A full description of the statistical parameters including central tendency (e.g. means) or other basic estimates (e.g. regression coefficient) AND variation (e.g. standard deviation) or associated estimates of uncertainty (e.g. confidence intervals)
☐	☒	For null hypothesis testing, the test statistic (e.g. *F*, *t*, *r*) with confidence intervals, effect sizes, degrees of freedom and *P* value noted<br>*Give P values as exact values whenever suitable.*
☐	☒	For Bayesian analysis, information on the choice of priors and Markov chain Monte Carlo settings
☒	☐	For hierarchical and complex designs, identification of the appropriate level for tests and full reporting of outcomes
☐	☒	Estimates of effect sizes (e.g. Cohen's *d*, Pearson's *r*), indicating how they were calculated

Our web collection on statistics for biologists contains articles on many of the points above.

Software and code

Policy information about availability of computer code

Data collection	NA
Data analysis	R (https://www.R-project.org/), plink (https://www.cog-genomics.org/plink2/) and APOE modeling source code (https://github.com/DRI-Cardiff/APOE-modelling); PRSice(https://www.prsice.info/), LDpred-inf(https://github.com/bvilhjal/ldpred), PRS-CS(https://github.com/getian107/PRScs), LDAK(http://dougspeed.com/ldak/)and SBayesR(https://cnsgenomics.com/software/gctb/Overview)

For manuscripts utilizing custom algorithms or software that are central to the research but not yet described in published literature, software must be made available to editors and reviewers. We strongly encourage code deposition in a community repository (e.g. GitHub). See the Nature Research guidelines for submitting code & software for further information.

Data

Policy information about availability of data

All manuscripts must include a data availability statement. This statement should provide the following information, where applicable:

- Accession codes, unique identifiers, or web links for publicly available datasets
- A list of figures that have associated raw data
- A description of any restrictions on data availability

Kunkle et al. 2019 summary statistics for the Stage 1 can be obtained from The National Institute on Aging Genetics of Alzheimer's Disease Data Storage Site (NIAGADS)—a NIA/NIH-sanctioned qualified-access data repository, under accession NG00075. UK Biobank data used in this study were available under UK Biobank approval (application 15175). Alzheimer's Disease Neuroimaging Initiative (ADNI) used in this study were available at the database (adni.loni.usc.edu), upon registration and compliance with the data usage agreement. ROSMAP, MSBB, MAYO datasets were available via Synapse platform (https://www.synapse.org: syn3191087, syn10901595, syn6101474, syn10901600, syn3817650, syn10901601). 1000 Genomes data are publically available and can be found at http://www.1000genomes.org. All HipSci data were accessed from http://www.hipsci.org (HipSci data access agreement 8759).

Field-specific reporting

Please select the one below that is the best fit for your research. If you are not sure, read the appropriate sections before making your selection.

- [x] Life sciences
- [ ] Behavioural & social sciences
- [ ] Ecological, evolutionary & environmental sciences

For a reference copy of the document with all sections, see nature.com/documents/nr-reporting-summary-flat.pdf

Life sciences study design

All studies must disclose on these points even when the disclosure is negative.

Sample size	1000 Genomes (503 individuals); UK Biobank 500,000 individuals; HipSci 1254 individuals, ADNI 770 individuals, ROSMAP 1196 individuals, MAYO 349 individuals, MSBB 349 individuals.
Data exclusions	The data exclusions were made by 1) genetics (standard quality control analysis) 2) overlap with samples in AD discovery summary statistics (Kunkle et al. 2019) and 3) if phenotypes did not meet clinical diagnosis of Alzheimer's Disease or cognitively normal status in case-control design.
Replication	NA
Randomization	NA
Blinding	NA

Reporting for specific materials, systems and methods

We require information from authors about some types of materials, experimental systems and methods used in many studies. Here, indicate whether each material, system or method listed is relevant to your study. If you are not sure if a list item applies to your research, read the appropriate section before selecting a response.

Materials & experimental systems

n/a	Involved in the study	
x		Antibodies
x		Eukaryotic cell lines
x		Palaeontology and archaeology
x		Animals and other organisms
x		Human research participants
x		Clinical data
x		Dual use research of concern

Methods

n/a	Involved in the study	
x		ChIP-seq
x		Flow cytometry
x		MRI-based neuroimaging
